# Plasmodesmata play a key role in leaf vein patterning

**DOI:** 10.1371/journal.pbio.3001806

**Published:** 2022-09-28

**Authors:** Leah R. Band

**Affiliations:** 1 Centre for Mathematical Medicine and Biology, School of Mathematical Sciences, University of Nottingham, Nottingham, United Kingdom; 2 Division of Plant and Crop Sciences, School of Biosciences, University of Nottingham, Sutton Bonington, United Kingdom

## Abstract

Leaf veins provide a vital transport route in plants, and the formation of vein patterns has fascinated many scientists over the years. This Primer explores a new PLOS Biology study which reveals how transport through plasmodesmata plays a key role in vein patterning.

## Teaser

Leaf veins provide a vital transport route in plants. The formation of leaf vein patterns has fascinated many scientists. In *PLOS Biology*, Linh and Scarpella reveal that transport through plasmodesmata plays a key role in vein patterning.

Plant veins play a vital role, providing an efficient transport route to transfer sugar, water, and nutrients throughout the plant. During leaf development, intricate patterns of veins form within a tissue of initially identical cells. How vein patterning is controlled has long fascinated scientists.

It is well established that the hormone auxin regulates vein patterning. The seminal studies of Sachs [1] showed that auxin application induces the formation of new veins; this observation led to the well-known “Canalisation hypothesis” that proposes that movement of an auxin-dependent signal through cells increases the cell’s capacity for auxin transport [1]. Later studies provided key insights into the Canalisation hypothesis by revealing that auxin carriers contribute to vein formation: As leaves develop, the PIN1 auxin efflux carrier becomes restricted to and polarised within the cell files that later become veins [2,3].

However, until now, the mechanistic basis of vein pattern formation remained elusive, given plants inhibited in both carrier-mediated auxin transport and auxin signalling still form some vein patterns [4]. In their new study published in *PLOS Biology*, Linh and Scarpella investigated whether diffusion through plasmodesmata plays a role in vein patterning, revealing this to be an essential component of vein patterning [5].

Plasmodesmata are membrane-lined pores that connect the cytoplasm of adjacent cells, enabling passive diffusion of water, nutrients, and other small molecules. Plasmodesmal fluxes can be modified by callose, which reduces plasmodesmal permeability by physically restricting the pore. Thus, plasmodesmata enable auxin diffusion across a tissue, which can be spatially and temporally modified by callose deposition. Such regulated plasmodesmal auxin diffusion has been shown to be important in phototropism, lateral root emergence, and leaf hyponasty [6–8].

**Fig 1:**
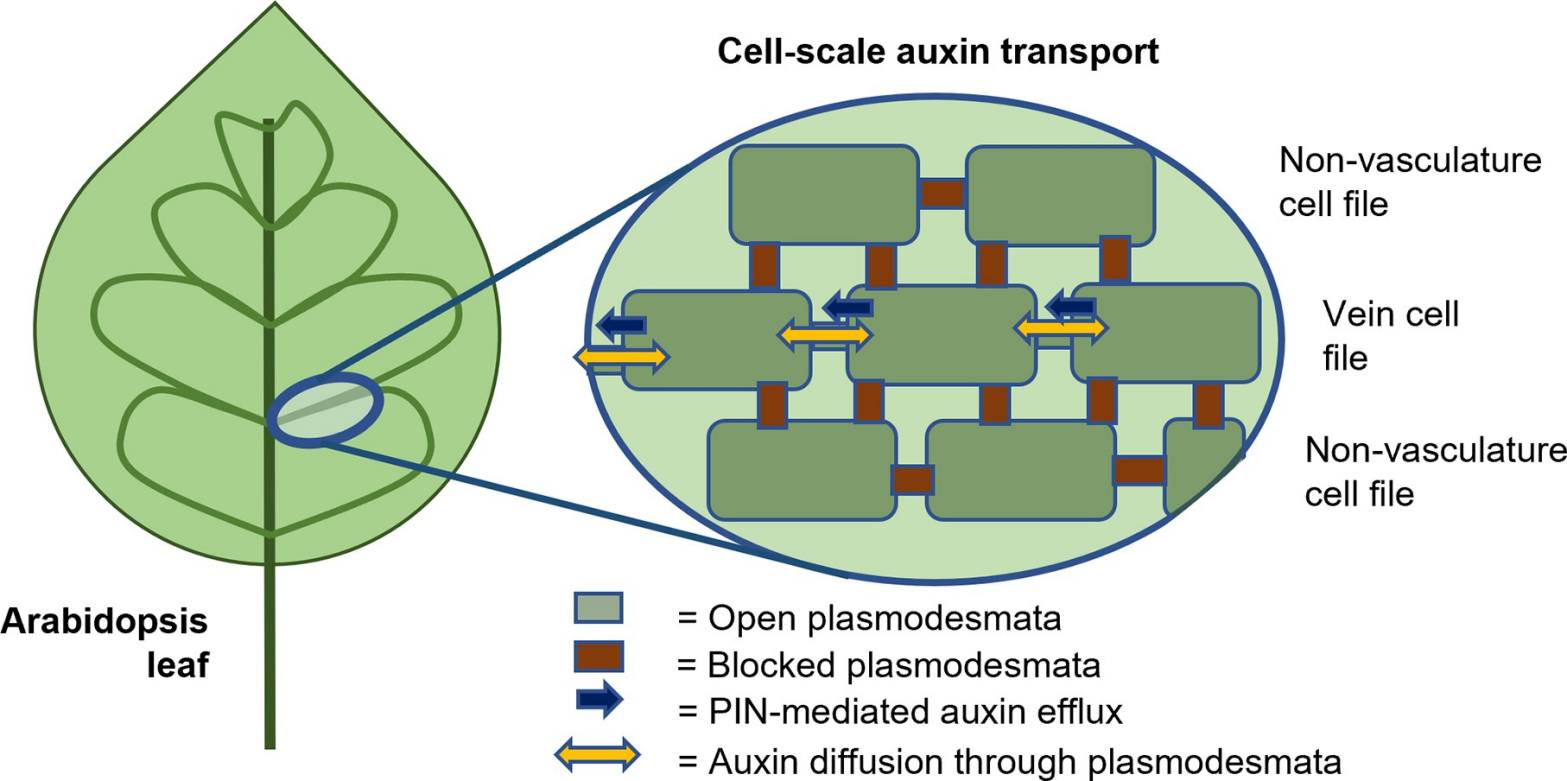
Schematic of key components of auxin transport governing leaf vein formation. Linh and Scarpella [5] show that in addition to polarised PIN1-mediated auxin transport within vein cell files, open plasmodesmata enable diffusion between adjacent vein cells. Their study reveals that diffusion of an auxin signal through plasmodesmata is essential to leaf vein patterning in *Arabidopsis*.

To test whether plasmodesmal diffusion also affects vein patterns, Linh and Scarpella [5] first modified plasmodesmal aperture using callose synthesis mutants (*cals3-d* and *gsl8*); in these mutants, they observed defects in vein patterning, with fewer veins, open loops, vein fragments, and vascular clusters. Linh and Scarpella then considered whether there are spatial and temporal variations in plasmodesmal permeabilities during vein development. By inducing and observing fluorescent proteins, they revealed that plasmodesmal permeability is high throughout the leaf at early stages; however, as the veins form, plasmodesmal diffusion reduces between the veins and non-vasculature tissue. Once developed, the veins are symplastically isolated, so that small molecules can diffuse through plasmodesmata between adjacent vein cells but cannot diffuse into the adjacent non-vein cells (Fig 1). These spatial differences in plasmodesmal diffusion were found to be essential for correct vein patterning.

Linh and Scarpella then considered whether plasmodesmal diffusion contributed to vein induction after auxin application and how this process co-ordinates with the established roles of auxin transport and signalling (described in [2–4]). Using the callose synthesis mutants (in which spatial variations in plasmodesmal permeability did not occur), auxin application induced veins in significantly fewer cases. Furthermore, applying auxin to wild-type leaves delayed the reduction in plasmodesmal permeability between the veins and non-vasculature tissue, suggesting a coupling between auxin levels and plasmodesmal fluxes.

The coupling between auxin and plasmodesmata was further investigated using chemical treatments: inhibiting polar auxin transport (by applying NPA) delayed the reduction in plasmodesmal permeability between veins and non-vasculature tissue, whereas inhibiting auxin-signalling (by applying PBA), promoted the restriction in plasmodesmal permeability. Thus, the regulation of plasmodesmal permeability depends on both polar auxin transport and auxin signalling. Furthermore, in the callose synthesis mutants, the connected and continuous PIN1 domains characteristic of vein patterning become gradually disconnected (due to loss of PIN1 expression in segments of the initial PIN1 domains), suggesting feedbacks between plasmodesmal and PIN1-mediated auxin transport.

Having established a crucial role for plasmodesmal diffusion in vein patterning, Linh and Scarpella questioned whether this process is regulated by GNOM (GN), which is a regulator of membrane trafficking and was shown to regulate an undiscovered component of vein patterning in [4]. They observed that regulation of plasmodesmal permeability is defective in *gn* mutants, and that simultaneous inhibition of the regulation of plasmodesmal permeability, auxin transport, and auxin signalling phenocopies the strong *gn* mutant. They concluded that GNOM (GN) forms a signalling hub controlling the 3 components of vein formation: auxin signalling, polar transport, and diffusion of an auxin signal through plasmodesmata.

Precisely how the plasmodesmal permeability is regulated during vein formation remains an open question. While the observations using callose synthesis mutants suggest that callose reduces plasmodesmal permeability, the data presented does not ruled out the possibility that differences in plasmodesmal density or structure also contribute to the observed spatial variations in plasmodesmal diffusion. Furthermore, the molecular details as to how auxin is regulating plasmodesmal permeabilities remain uncovered. Key to previous studies on phototropism and lateral root emergence [6,8] was the observation that auxin increases callose levels to restrict its own movement. In contrast, a study of leaf hyponasty found that the effective plasmodesmal permeability was not affected by exogeneous auxin treatments [7]. However, this new study on vein patterning provides a further scenario, with plasmodesmal diffusion being high between 2 adjacent vein cells with high auxin, but low between a high-auxin vein cell and adjacent low-auxin non-vasculature cell. Precisely how auxin regulates these plasmodesmal permeabilities, how GNOM contributes to this regulation, and how the plasmodesmal regulation combines with auxin’s regulation of PIN1 levels [9] remain intriguing avenues for future research.

In summary, Linh and Scarpella [5] elucidate a key mechanistic component of Sach’s Canalisation hypothesis. Their elegant study demonstrates that regulation of plasmodesmata is essential for vein patterning. They conclude that vein patterning occurs through the co-ordinated action of auxin signalling, polar transport, and diffusion of an auxin signal through plasmodesmata, 3 processes that are all regulated by GNOM. How these findings translate to either other plant patterning processes or vein patterning in other species [10] form open and exciting questions for future research.
